# Dose‐dependent effect of mesenchymal stromal cells co‐grafted with dopaminergic neurons in a Parkinson's disease rat model

**DOI:** 10.1111/jcmm.16900

**Published:** 2021-09-18

**Authors:** Jannette Rodriguez‐Pallares, Maria Garcia‐Garrote, Juan A. Parga, Jose Luis Labandeira‐Garcia

**Affiliations:** ^1^ Cellular and Molecular Neurobiology of Parkinson's disease Research Center for Molecular Medicine and Chronic diseases (CIMUS) IDIS University of Santiago de Compostela Santiago de Compostela Spain; ^2^ Networking Research Center on Neurodegenerative Diseases (CIBERNED) Madrid Spain

**Keywords:** cell death, cell therapy, dopaminergic neuron, Parkinson, stem cells, transplantation

## Abstract

A major limiting factor for cell therapy in Parkinson's disease is the poor survival and reinnervation capacity of grafted dopaminergic neurons, independently of the cell source. Mesenchymal stromal cells (MSCs) have high capability to regulate the local environment through the release of trophic, antiapoptotic and immunomodulatory factors. In this work, we investigated whether co‐grafting of MSCs could improve the survival and reinnervation ability of dopaminergic precursors transplanted in animal models of Parkinson's disease. Rats with total unilateral dopaminergic denervation were grafted with a cell suspension of rat dopaminergic precursors (500,000 cells) with or without a high (200,000 cells) or low (25,000 cells) number of MSCs. Eight weeks after grafting, rats were tested for motor behaviour and sacrificed for histological analysis. Our results showed that the survival of dopaminergic neurons and graft‐derived striatal dopaminergic innervation was higher in rats that received co‐grafts containing a low number of MSCs than in non‐co‐grafted controls. However, the survival of dopaminergic neurons and graft‐derived dopaminergic reinnervation was lower in rats receiving co‐grafts with high number of MSCs than in non‐co‐grafted controls. In conclusion, co‐grafting with MSCs or MSCs‐derived products may constitute a useful strategy to improve dopaminergic graft survival and function. However, a tight control of MSCs density or levels of MSCs‐derived products is necessary.

## 1 INTRODUCTION

Parkinson's disease (PD) is a neurodegenerative disorder caused by the selective degeneration of dopaminergic cells in the substantia nigra leading to major problems in the motor system. One promising therapeutic approach for PD is dopaminergic cell‐replacement therapy, in which dopaminergic precursors are grafted into the striatum to restore the lost dopaminergic neurotransmission. Previous clinical trials based on foetal dopamine neuron transplantation have shown promising results,^1^ but also significant limitations including the survival of grafted dopaminergic neurons, which is very poor independent of the source of dopaminergic neurons.^2^, ^3^ Dopaminergic cells from induced pluripotent stem cells (iPSCs) or embryonic stem cells are being investigated as alternative cell sources for PD patients.^4^, ^5^ However, strategies to improve the survival of grafted cells and improve host reinnervation must be developed.

Mesenchymal stromal cells (MSCs) are multipotent cells originally isolated from the stroma of the bone marrow.^6^ Different studies have shown their capability to regulate the local environment through the release of immunomodulatory, antiapoptotic and trophic factors. These properties make them an attractive cell source for repairing damaged tissue.^7^ Consistent with this, we have previously observed that conditioned medium derived from bone marrow MSCs increases the viability of dopaminergic cells of rat and human origin in cultures.^8^ However, possible benefits of MSCs on dopaminergic graft survival have not been investigated. In this work, we used mixed suspensions of dopaminergic cells and MSCs (ie co‐grafts) in a rat model of PD to study the possible positive effects of MSCs on survival and functionality of grafted dopaminergic neurons and their possible use for improving the results of transplantation therapy in PD.

## 2 MATERIALS AND METHODS

Young adult rats were subjected to maximal unilateral dopaminergic denervation with 6‐hydroxydopamine (6‐OHDA). One month after 6‐OHDA injections, rats with maximal lesions (dopaminergic depletion >90%) were identified in a rotometer and confirmed with the cylinder test. One week later, rats showing behavioural criteria for maximal dopaminergic denervation were selected for transplantation (ie to investigate the effect of MSCs on the survival of grafted dopaminergic precursors). In a first series of experiments, rats with maximal dopaminergic denervation were grafted with dopaminergic precursors derived from foetal ventral mesencephalon (500,000 cells; 13 days of gestation, E13; VM; *n* = 5 rats, control group) or co‐grafted (*n* = 6 rats) with 500,000 cells from the same VM suspension and a high number (200,000 cells) of MSCs (VM + high MSC‐group). The number of MSCs was determined on the basis of previous studies (see Appendix S1). In a second series of experiments, rats with maximal dopaminergic denervation were grafted with 500,000 cells from a VM suspension (*n* = 5 rats; control group) or co‐grafted (*n* = 5 rats) with 500,000 cells from the same VM suspension and a low number (25,000 cells) of MSCs (VM + low MSC‐group). MSCs were from femur bone marrow and labelled with the live‐cell fluorescent dye Cell TrackerTM Orange (CMTMR, Thermo Fisher Scientific). Eight weeks after grafting, the rats were tested again in the cylinder and the rotometer and then killed for histological analysis. Rats from each series (co‐grafts and the corresponding controls) were always processed in the same batch. Additional details on materials and methods are provided as supplementary information (see Appendix S1).

## 3 RESULTS

### 3.1 Effects of co‐grafting of VM cell suspensions and a high number of MSCs

**FIGURE 1:**
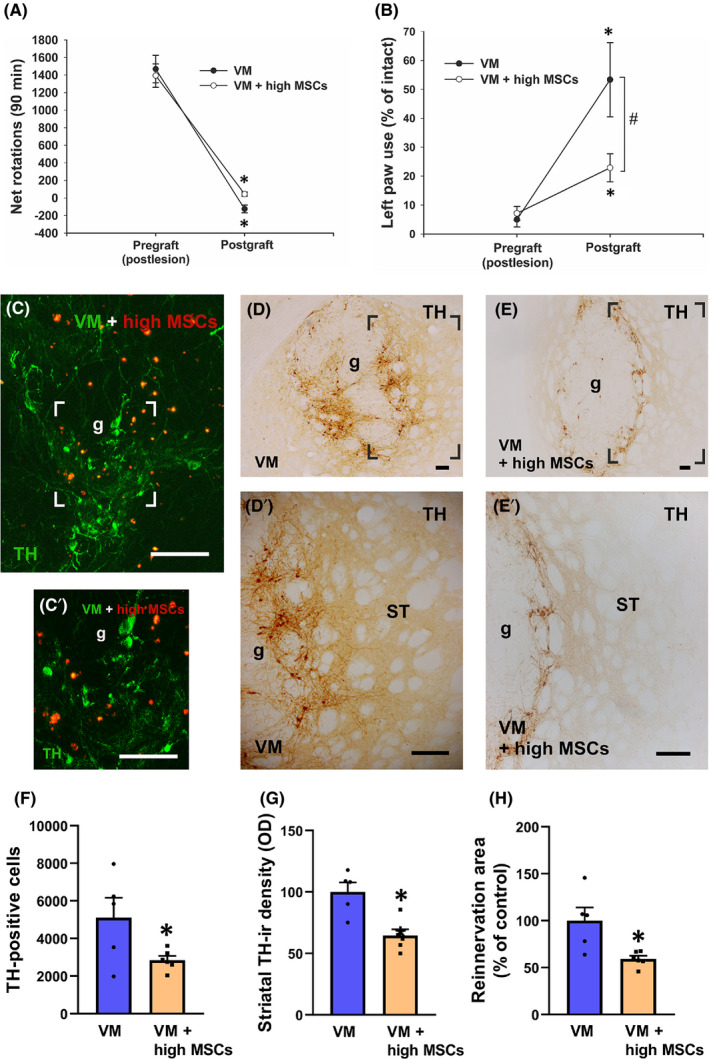
Behavioural (A, B) and histological (C–H) analysis of co‐grafts of ventral mesencephalic (VM) cell suspensions and a high number of mesenchymal stromal cells (MSCs). Rotational values (A) for both grafted groups did not differ significantly between groups. In the cylinder test (B), VM‐grafted rat scores were significantly better than in the VM + high MSC‐grafted group. Immunoreactivity for tyrosine hydroxylase (TH) in rats subjected to dopaminergic denervation and co‐grafting (C–C′, E–E′; *n* = 6) or grafted with VM cells alone (D–D′; *n* = 5), and showing green fluorescence from TH‐ir dopaminergic cells and red fluorescence from grafted MSCs (C–C′). Areas boxed in C–E are magnified in C′–E′. F, Number of TH‐positive (dopaminergic) neurons in both groups. G, Density of TH‐immunoreactive (‐ir) fibres in the reinnervation area, estimated as optical density (OD) and expressed as a percentage of the value obtained in the control group (VM‐grafted group). H, Reinnervation area (ie striatal host area reinnervated by graft‐derived TH‐ir fibres) in the mentioned groups. Data represent mean ± standard error of the mean (SEM). In A, B, *p < 0.05 vs lesioned rats (ie postlesion and pregrafting) and ^#^ *p* < 0.05 between grafted groups (ie VM and VM + high MSCs) (Student's *t* test). In F–G, *p < 0.05 vs VM‐grafted rats (Student's *t* test). Scale bar = 100 μm and 50 μm for C′. ST, striatum; g, graft

Drug‐induced rotational behaviour was used as an *in vivo* indicator of graft survival. The lesion induced a strong rotational asymmetry, with ipsilateral turning (ie towards the denervated side) after amphetamine administration, both in VM‐grafted rats (1468.2 ± 156.0 turns) and in VM + high MSC‐grafted rats (1393.7 ± 133.8 turns) (Figure 1A). Eight weeks after transplantation, both groups of grafted rats (ie VM and VM + high MSCs) showed a marked decrease in amphetamine‐induced rotations (−124.8 ± 44.4 turns in VM‐grafted animals and 44.5 ± 23.7 in VM + high MSC‐grafted rats) (Figure 1A). The cylinder test was used to obtain further information on behavioural recovery (Figure 1B). In the cylinder test, spontaneous paw use is approximately 50% for each of the forepaws in non‐lesioned controls. After 6‐OHDA lesion, the use of the left/impaired forepaw was reduced to 5.0 ± 2.6% in the VM‐grafted group and 7.1 ± 2.4% in the VM + high MSC‐grafted group (Figure 1B). Eight weeks after transplantation, both groups of grafted rats showed a significant improvement in use of the lesioned forepaw, although the improvement was significantly lower in VM + high MSC‐grafted rats (53.3 ± 12.8% left paw use in the VM‐grafted group and 22.9 ± 4.9% in the VM + high MSC‐grafted group) (Figure 1B).

As detailed in previous studies,^9^ intrastriatal grafts of foetal VM contained numerous TH‐immunoreactive (‐ir) neurons, which were not evenly distributed within the graft. Most cells were grouped in patches located at the periphery of the graft, whereas the central area of the grafts was usually TH‐negative (Figure 1C,D,E,C′,D′,E′). Cells showing red fluorescence revealed the presence of MSCs in the grafts (Figure 1C,C′). Surprisingly, the total number of TH‐ir neurons was significantly lower in VM + high MSC‐grafted rats than in the rats transplanted with VM tissue alone (Figure 1F). However, the average graft volume in the VM + high MSC‐grafted group was not significantly different from that observed in VM‐grafted rats (0.351 ± 0.023 mm^3^ and 0.454 ± 0.065 mm^3^, respectively). The density of TH‐ir fibres in the reinnervation area (Figure 1G) and the size of the reinnervation area (Figure 1H) were reduced in the VM + high MSC‐grafted group compared to VM‐grafted group. Altogether (ie similar graft volume with decrease in TH‐ir neurons and fibres) suggests an increase in selective loss of dopaminergic neurons in the VM + high MSC‐grafted group.

### 3.2 Effects of co‐grafting of VM cell suspensions and a low number of MSCs

**FIGURE 2:**
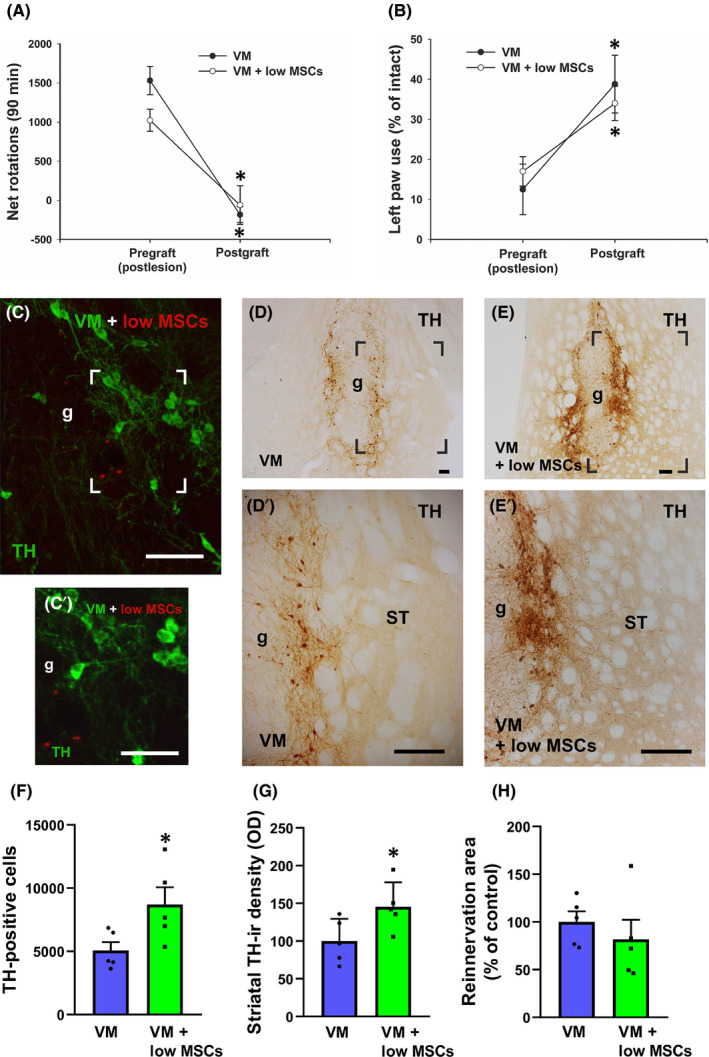
Behavioural (A, B) and histological (C–H) analysis of co‐grafts of ventral mesencephalic (VM) cell suspensions and a low number of mesenchymal stromal cells (MSCs). Rotational values (A) and performance of the cylinder test (B) did not differ significantly between groups. Immunoreactivity for tyrosine hydroxylase (TH) in rats subjected to dopaminergic denervation and co‐grafting (C–C′, E–E′; *n* = 5) or grafted with VM cells alone (D–D′; *n* = 5), and showing green fluorescence from TH‐ir dopaminergic cells and red fluorescence from grafted MSCs (C–C′). Areas boxed in C–E are magnified in C′–E′. F, Number of dopaminergic (TH‐positive) neurons) in both groups. G, Density of TH‐immunoreactive (‐ir) fibres in the reinnervation area, estimated as optical density (OD) and expressed as a percentage of the value obtained in the control group (VM‐grafted group). H, Reinnervation area (ie striatal host area reinnervated by graft‐derived TH‐ir fibres) in both groups. Data represent mean ± standard error of the mean (SEM). In A, B, *p < 0.05 vs lesioned rats (ie postlesion and pregrafting), and ^#^ *p* < 0.05 between grafted groups (ie VM and VM + low MSCs) (Student's *t* test). In F–G, *p < 0.05 vs control VM‐grafted rats (Student's *t* test). Scale bar = 100 μm and 50 μm for C′. ST, striatum; g, graft

As described above, dopaminergic lesion induced a strong rotational asymmetry, and amphetamine‐induced ipsilateral turning (ie towards the denervated side) both in the VM‐grafted group (1530.8 ± 179.9 turns) and in VM + low MSC‐grafted group (1023.4 ± 141.5 turns) (Figure 2A). Amphetamine‐induced rotational behaviour was completely reverted by grafts, both in rats receiving VM transplants and rats co‐grafted with VM + low number of MSCs (−182.5 ± 100.7 turns and −60.4 ± 246.0 turns, respectively) (Figure 2A). After the 6‐OHDA lesion, the use of the left/impaired forepaw was reduced to 12.5 ± 6.3% in the VM‐grafted group and 17.0 ± 3.7% in the VM + low MSC‐grafted group (Figure 2B) in the cylinder test. After grafting, a significant improvement in the cylinder test was observed in both groups of transplanted rats (Figure 2B) although no significant difference in the recovery was detected between both groups using this test (38.8 ± 7.2% left paw use in the VM‐grafted group and 34.0 ± 4.3% in the VM + low MSC‐grafted group).

Histological analysis showed that the total number of TH‐ir neurons was significantly higher in animals transplanted with VM + low MSC than in VM‐grafted animals (Figure 2C–F and C′–E′). Cells showing red fluorescence revealed the presence of MSCs in the grafts (Figure 2C,C′). No significant difference in the volume of the graft was observed between groups (0.455 ± 0.064 mm^3^ in VM‐grafted rats and 0.510 ± 0.089 mm^3^ in VM + low MSC‐grafted rats). However, the density of TH‐ir fibres in the reinnervation area was higher in the rats that received co‐grafts of VM and low number of MSCs compared to animals that received suspensions of VM cells alone (Figure 2G). The size of the reinnervation area in the VM + low MSC‐grafted group was similar to that observed in the VM‐grafted group (Figure 2H). Altogether (ie a similar graft volume with increase in TH‐ir neurons and fibres) suggests a decrease in selective loss of dopaminergic neurons in the VM + low MSC‐grafted group. The lack of significant differences in motor behaviour suggests that the observed histological improvement is not enough to induce functional differences or, more probably, that the sensitivity of these basic behavioural tests is not sufficient to detect a difference in the behavioural improvement.

## 4 DISCUSSION

The lack of alternative sources to dopaminergic neuroblasts from the foetal substantia nigra and the high rate of dopaminergic cell loss during the transplantation procedure and the early post‐transplantation period have been two major limitations for cell therapy as possible strategy for PD treatment. Recent advances with alternative sources of dopaminergic neurons such as iPSCs and embryonic stem cell dopaminergic differentiation have led to the beginning of several promising clinical trials.^4^, ^5^ In addition to a number of specific limitations related to transplantation of iPSCs or embryonic‐derived dopaminergic neurons,^5^, ^10^ the poor survival and integration with the host brain observed in grafts of foetal neuroblasts remain or is even more problematic in the case of grafts from the above‐mentioned alternative dopaminergic neuron sources.^1^, ^11^

Major factors responsible for the high levels of dopaminergic cell loss during the transplantation process and early post‐transplantation period have not been totally clarified. However, grafting‐related cell trauma, lack of growth factors, poor vascularization, neuroinflammation and other factors have been involved.^12^, ^13^ MSCs have been effective against several of the above‐mentioned factors,^7^, ^8^ and a neuroprotective effect on grafted dopaminergic neurons could be expected. The number of grafted MSCs may decrease over the time,^14^, ^15^ and part of the CMTMR‐labelled cells may correspond to death MSCs phagocyted by glial cells. However, it is well known that practically all the dopaminergic cell loss occurs during the first few days after the transplantation,^16^, ^17^ when most grafted MSCs are present. In the present study, we showed the capacity of co‐grafted MSCs to induce beneficial effects on survival and reinnervation ability of grafted dopaminergic neurons. Surprisingly, the increase in the number of co‐grafted MSCs led to detrimental effects. The mechanisms responsible for this effect are still unclear. A high concentration of MSCs may induce MSC senescence, damaged mitochondrial transfer or dysregulation of pro‐inflammatory cytokine production.^18^, ^19^, ^20^ In any case, the results reveal that further studies are necessary to know the exact proportion of dopaminergic/MSCs that leads to the highest neuroprotective effects. In addition, future studies are necessary to identify MSCs‐derived products that are responsible for the positive effects and may be used as a substitute for MSCs. However, the present results suggest that the dose must be tightly adjusted to the number of grafted dopaminergic cells.

## CONFLICT OF INTEREST

The authors declare that there are no competing interests associated with the manuscript.

## AUTHOR CONTRIBUTIONS

**Jannette Rodriguez‐Pallares:** Conceptualization (equal); investigation (equal); writing–review and editing (equal). **Maria Garcia‐Garrote:** Formal analysis (equal); investigation (equal); methodology (equal); writing–review and editing (equal). **Juan A. Parga:** Data curation (equal); formal analysis (equal); investigation (equal); writing–review and editing (equal). **Jose Luis Labandeira‐Garcia:** Conceptualization (equal); formal analysis (equal); supervision (equal); writing–review and editing (equal).

## Supporting information

Appendix S1

## Data Availability

Data are available from the corresponding authors upon reasonable request.

## References

[1] Bjorklund A , Lindvall O . Replacing dopamine neurons in Parkinson's disease: how did it happen? J Parkinsons Dis. 2017;7(s1):S21‐S31. 28282811 10.3233/JPD-179002 PMC5345652

[2] Rylander D , Bagetta V , Pendolino V , et al. Region‐specific restoration of striatal synaptic plasticity by dopamine grafts in experimental Parkinsonism. Proc Natl Acad Sci U S A. 2013;110(46):4375. 10.1073/pnas.1311187110 PMC3831970 24170862

[3] Thompson L , Bjorklund A . Survival, differentiation, and connectivity of ventral mesencephalic dopamine neurons following transplantation. Prog Brain Res. 2012;200:61‐95. 23195415 10.1016/B978-0-444-59575-1.00004-1

[4] Piao J , Zabierowski S , Dubose BN , et al. Preclinical efficacy and safety of a human embryonic stem cell‐derived midbrain dopamine progenitor product, MSK‐DA01. Cell Stem Cell. 2021;28(2):217‐229.e7. 33545080 10.1016/j.stem.2021.01.004 PMC7903922

[5] Takahashi J . iPS cell‐based therapy for Parkinson's disease: a Kyoto trial. Regen Ther. 2020;13:18‐22. 33490319 10.1016/j.reth.2020.06.002 PMC7794047

[6] Viswanathan S , Shi Y , Galipeau J , et al. Mesenchymal stem versus stromal cells: International society for cell & gene therapy (ISCT(R)) mesenchymal stromal cell committee position statement on nomenclature. Cytotherapy. 2019;21(10):1019‐1024. 31526643 10.1016/j.jcyt.2019.08.002

[7] Fricova D , Korchak JA , Zubair AC . Challenges and translational considerations of mesenchymal stem/stromal cell therapy for Parkinson's disease. NPJ Regen Med. 2020;5(1):20‐y. 10.1038/s41536-020-00106-y PMC7641157 33298940

[8] Parga JA , Garcia‐Garrote M , Martinez S , Raya A , Labandeira‐Garcia JL , Rodriguez‐Pallares J . Prostaglandin EP2 receptors mediate mesenchymal stromal cell‐neuroprotective effects on dopaminergic neurons. Mol Neurobiol. 2018;55(6):4763‐4776. 28717970 10.1007/s12035-017-0681-5

[9] Tiklová K , Nolbrant S , Fiorenzano A , et al. Single cell transcriptomics identifies stem cell‐derived graft composition in a model of Parkinson's disease. Nat Commun. 2020;11(1):2434. 32415072 10.1038/s41467-020-16225-5 PMC7229159

[10] Adler AF , Cardoso T , Nolbrant S , et al. hESC‐derived dopaminergic transplants integrate into basal ganglia circuitry in a preclinical model of Parkinson's disease. Cell Rep. 2019;28(13):3462‐3473.e5. 31553914 10.1016/j.celrep.2019.08.058 PMC6899556

[11] Jang SE , Qiu L , Chan LL , Tan EK , Zeng L . Current status of stem cell‐derived therapies for Parkinson's disease: From cell assessment and imaging modalities to clinical trials. Front Neurosci. 2020;14:558532. 33177975 10.3389/fnins.2020.558532 PMC7596695

[12] Winkler C , Kirik D , Bjorklund A . Cell transplantation in Parkinson's disease: how can we make it work? Trends Neurosci. 2005;28(2):86‐92. 15667931 10.1016/j.tins.2004.12.006

[13] Gantner CW , de Luzy IR , Kauhausen JA , et al. Viral delivery of GDNF promotes functional integration of human stem cell grafts in Parkinson's disease. Cell Stem Cell. 2020;26(4):511‐526.e5. 32059808 10.1016/j.stem.2020.01.010

[14] Moloney TC , Dockery P , Windebank AJ , Barry FP , Howard L , Dowd E . Survival and immunogenicity of mesenchymal stem cells from the green fluorescent protein transgenic rat in the adult rat brain. Neurorehabil Neural Repair. 2010;24(7):645‐656. 20378924 10.1177/1545968309357745

[15] Offen D , Barhum Y , Levy YS , et al. Intrastriatal transplantation of mouse bone marrow‐derived stem cells improves motor behavior in a mouse model of Parkinson's disease. J Neural Transm Suppl. 2007;72:133‐143. 10.1007/978-3-211-73574-9_16 17982886

[16] Emgard M , Hallin U , Karlsson J , Bahr BA , Brundin P , Blomgren K . Both apoptosis and necrosis occur early after intracerebral grafting of ventral mesencephalic tissue: a role for protease activation. J Neurochem. 2003;86(5):1223‐1232. 12911630 10.1046/j.1471-4159.2003.01931.x

[17] Sortwell CE , Pitzer MR , Collier TJ . Time course of apoptotic cell death within mesencephalic cell suspension grafts: implications for improving grafted dopamine neuron survival. Exp Neurol. 2000;165(2):268‐277. 10993687 10.1006/exnr.2000.7476

[18] Ding Y , Liang X , Zhang Y , et al. Rap1 deficiency‐provoked paracrine dysfunction impairs immunosuppressive potency of mesenchymal stem cells in allograft rejection of heart transplantation. Cell Death Dis. 2018;9(3):386. 29515165 10.1038/s41419-018-0414-3 PMC5842217

[19] Mahrouf‐Yorgov M , Augeul L , Da Silva CC , et al. Mesenchymal stem cells sense mitochondria released from damaged cells as danger signals to activate their rescue properties. Cell Death Differ. 2017;24(7):1224‐1238. 28524859 10.1038/cdd.2017.51 PMC5520168

[20] Zhang Y , Yu Z , Jiang D , et al. PSC‐MSCs with high intrinsic MIRO1 and sensitivity to TNF‐α yield efficacious mitochondrial transfer to rescue anthracycline‐induced cardiomyopathy. Stem Cell Reports. 2016;7(4):749‐763. 27641650 10.1016/j.stemcr.2016.08.009 PMC5063626

