# Supplementary material for: Dose‐dependent effect of mesenchymal stromal cells co‐grafted with dopaminergic neurons in a Parkinson's disease rat model
Source: J Cell Mol Med. 2021 Sep 18;25(20):9884–9. doi: 10.1111/jcmm.16900 (PMC8505847; doi:10.1111/jcmm.16900)
Supplement: Supplementary file 1 — Appendix S1 [file JCMM-25-9884-s001.pdf]

Supplemental data

Materials and Methods

Animal models

Eight week female Sprague–Dawley rats (~250 g at the beginning of the study) were used. All experiments were carried out in accordance with the Spanish (RD53/2013) and European Communities Council Directive (2010/63/EU and 86/609/CEE) and were approved by the corresponding committees at the University of Santiago de Compostela and Galician Government (15005/15/002).

Mesenchymal stromal cell culture

Mesenchymal stromal cells (MSCs) were harvested from femur bone marrow of 6-8-week-old Sprague Dawley rats as described previously[1]. Cells were plated at 5,000 cells/cm^2 onto 75 cm^2 flasks and maintained in a humidified CO_2 incubator (5% CO_2; 37ºC). Culture medium was replaced every 3-4 days, and the cells were passaged 4 times before being used for experiments. The phenotype and multipotentiality of MSCs were determined as described previously[2].

Prior to grafting, MSCs were labelled with the live-cell fluorescent dye Cell Tracker^{TM} Orange (CMTMR; 15 μM; Invitrogen, Thermo Fisher Scientific) according to the manufacturer's instructions. Briefly, the culture medium was removed and pre-warmed dye working solution in Dulbecco's modified Eagle's medium (DMEM; Gibco, Thermo Fisher Scientific) was added to the cells for 30 min. The dye solution was then replaced with fresh culture medium and cells were incubated for another 30 min under cell growth conditions. Finally, the medium was removed and the cells were washed

with phosphate buffer saline (PBS) before dissociating with TrypLE (Gibco, Thermo Fisher Scientific). The enzymatic activity was neutralized with fresh DMEM with 10% fetal bovine serum (FBS; Corning, Thermo Fisher Scientific) and the cells were centrifuged to pellet and resuspended before grafting.

6-OHDA lesion and transplantation surgery

Unilateral lesions of the dopaminergic system were performed by injection into the right medial forebrain bundle of 12 μg of 6-OHDA HBr (in 4 μl of sterile saline containing 0.2% ascorbic acid; Sigma-Aldrich). The stereotaxic coordinates were[3]: 3.7 mm posterior to the bregma, -1.6 mm lateral to midline and 8.8 mm ventral to the skull, with the tooth bar set at -3.3 mm. VM cell suspensions were prepared from fetal (13 days of gestation, E13) VM[3,4]. The pieces of VM were dissected out and incubated in 0.1% trypsin (Sigma-Aldrich), 0.05% DNase (Sigma-Aldrich) and DMEM (Gibco, Thermo Fisher Scientific) for 20 min at 37°C. The tissue was then rinsed in DNase/DMEM and mechanically dissociated to obtain a cell suspension with a final concentration of about 165,000 cells/μl. The control groups of rats were grafted with 500,000 VM cells (in 3μl) and MSC medium (3μl). The rats implanted with co-grafts received 500,000 VM cells (3μl) from the same suspension of the corresponding control group and 200,000 MSCs (VM+high MSC-group; 3μl), or 25,000 MSCs (VM+low MSC-group; 3μl). An amount of 200,000 MSCs is the most common number of MSCs implanted in the striatum, showing effects of neuroprotection/rescue of the degenerating dopaminergic system.[5,6] However, grafts of 25-30,000 MSCs have also been shown functional effects in other studies.[7] The corresponding cell suspension was slowly (10 min) administered to each rat into the striatum at a single injection site (anterior to bregma = 0.6 mm, lateral to midline = -2.7 mm, ventral to the dura = 4.5 mm, with the

tooth bar set at 0 mm). As it is known that the viability of the dopaminergic cells in the VM suspension decreases over time, the grafts were performed in two sessions (a first series and a second series of experiments) using new cell suspensions for the VM + low MSC-group and VM + high MSC-group, which were compared with the corresponding controls of rats grafted with the same VM suspension. In addition, within each grafting session, control (VM) and treated (VM+MSCs) rats were grafted alternately to minimize effect of time on the dopaminergic cell viability.

Behavioral testing of lesion efficacy and graft survival

The efficacy of the lesion and graft survival was evaluated one month after lesion and 8 weeks after grafting by the rotometer test[8] and the cylinder test[9]. The efficacy of the lesion and graft survival was also confirmed by subsequent tyrosine hydroxylase (TH; as dopaminergic marker) immunohistochemistry of the substantia nigra and striatum at the end of the experiments (see below). Drug-induced rotation was tested in a bank of eight automated rotometer bowls (Rota-count 8, Columbus Instruments, Columbus, OH, USA). Turning behavior was monitored for 90 min after injection of D-amphetamine (5 mg/kg; intraperitoneally; Sigma-Aldrich). Rats showing at least 7 ipsiversive turns per minute after injection of amphetamine were used. Spontaneous forelimb use was assessed by an observer blinded to the identity of the animals, who scored the number of forelimb contacts with the cylinder wall, recording a total number of 20 touches for each animal. Forelimb asymmetry, assessed as use of the left impaired paw, was expressed as percentage of the total number of touches.

Immunolabelling and quantitative histological analysis

Animals were deeply anesthetized and perfused with cold 4% paraformaldehyde in 0.1 M phosphate buffer pH 7.4. Brains were dissected out, cryoprotected in the same buffer containing 30% sucrose and cut with a microtome into 40 μm-thick coronal sections. Series of free-floating sections were stained using a mouse monoclonal antibody to TH. Sections were preincubated for 1 h in a blocking solution containing 10% normal serum with 0.25% Triton™ X-100 (Sigma-Aldrich) in 0.02 M potassium phosphate-buffered saline containing 1% bovine serum albumin (KPBS-BSA) at room temperature. The sections were then incubated overnight at 4°C with the corresponding primary antibody anti-TH (1:10,000; Sigma-Aldrich). The sections were subsequently incubated, first for 1 h with the corresponding biotinylated secondary antibody (horse anti-mouse; 1:500; Vector Laboratories) and then for 90 min with an avidin–biotin–peroxidase complex (ABC complex; 1:150; Vector Laboratories). The labeling was visualized using 3,3´diaminobenzidine (DAB; Sigma-Aldrich)[3,4].

Selected striatal sections were processed for TH immunofluorescence. Briefly, sections were incubated overnight at 4ºC with the corresponding TH primary antibody at double concentration (see above). After rinsing, the sections were incubated for 180 min with Alexa Fluor 488-conjugated donkey anti-mouse IgG (1: 200; Molecular Probes; Thermo Fisher Scientific). To visualize the fluorescent labeling, we used an inverted microscope equipped with ApoTome system (Zeiss, Oberkochen, Germany).

Quantification of TH-immunoreactive (-ir) neurons in grafts was carried out in every fourth section to cover the entire graft from the rostral tip to the caudal end. Sampling was carried out with the Computer Assisted Stereological Toolbox (CASTGrid system; Olympus, Ballerup, Denmark) and the observed was blind. The total number of TH-positive neurons in the grafts were calculated according to the optical fractionator formula[10]. A counting frame (1800 μm^2) was placed at random on

the first counting area and systematically moved through all counting areas until the entire delineated area was sampled. Neuron profiles were observed with a 100x oil objective (NA 1.4). The graft volume was estimated according to Cavalieri's method[11]. The striatal graft-derived reinnervation area (TH-ir area surrounding the graft) was measured using the CASTGrid system. At least four sections through the grafted striatum of each rat were measured and expressed in mm^2. The density of the TH-ir fibers in the graft-derived reinnervation area was estimated as the optical density of striatal TH-ir with the aid of NIH-Image 1.55 image analysis software. At least three sections through the graft of each animal were measured, and for each section optical density was corrected by subtraction of background as observed in the corpus callosum.

Statistical Analysis

Two group's comparisons (i.e. co-graft and the corresponding VM control) were analyzed by the Student's t-test. The results are presented as means ± standard error of the mean (SEM). Differences at $p < 0.05$ were considered as statistically significant. All statistical analyses were performed with SigmaPlot 11.0 (Systat Software Inc.).

References

1. Jones J, Jaramillo-Merchan J, Bueno C, Pastor D, Viso-Leon M, Martinez S. Mesenchymal stem cells rescue Purkinje cells and improve motor functions in a mouse model of cerebellar ataxia. *Neurobiol Dis*. 2010;40(2):415-423.

2. Parga JA, Garcia-Garrote M, Martinez S, Raya A, Labandeira-Garcia JL, Rodriguez-Pallares J. Prostaglandin EP2 receptors mediate mesenchymal stromal cell-

neuroprotective effects on dopaminergic neurons. *Mol Neurobiol*. 2018;55(6):4763-4776.

3. Rodriguez-Pallares J, Joglar B, Munoz-Manchado AB, Villadiego J, Toledo-Aral JJ, Labandeira-Garcia JL. Cografting of carotid body cells improves the long-term survival, fiber outgrowth and functional effects of grafted dopaminergic neurons. *Regen Med*. 2012;7(3):309-322.

4. Rodriguez-Pallares J, Rodriguez-Perez AI, Munoz A, Parga JA, Toledo-Aral JJ, Labandeira-Garcia JL. Effects of rho kinase inhibitors on grafts of dopaminergic cell precursors in a rat model of Parkinson's disease. *Stem Cells Transl Med*. 2016;5(6):804-815.

5. Hoban DB, Howard L, Dowd E. GDNF-secreting mesenchymal stem cells provide localized neuroprotection in an inflammation-driven rat model of Parkinson's disease. *Neuroscience* 2015;303:402-411.

6. Mendes-Pinheiro B, Anjo SI, Manadas B, et al. Bone Marrow Mesenchymal Stem Cells' Secretome Exerts Neuroprotective Effects in a Parkinson's Disease Rat Model. *Front Bioeng Biotechnol*. 2019;7:294.

7. Cova L, Armentero MT, Zennaro E, et al. Multiple neurogenic and neurorescue effects of human mesenchymal stem cell after transplantation in an experimental model of Parkinson's disease. *Brain Res*. 2010;1311:12-27.

8. Hudson JL, van Horne CG, Stromberg I, et al. Correlation of apomorphine- and amphetamine-induced turning with nigrostriatal dopamine content in unilateral 6-hydroxydopamine lesioned rats. *Brain Res*. 1993;626(1-2):167-174.

9. Schallert T, Kozlowski DA, Humm JL, Cocke RR. Use-dependent structural events in recovery of function. *Adv Neurol*. 1997;73:229-238.

10. West MJ, Slomianka L, Gundersen HJ. Unbiased stereological estimation of the total number of neurons in thesubdivisions of the rat hippocampus using the optical fractionator. *Anat Rec*. 1991;231(4):482-497.

11. Gundersen HJ, Bendtsen TF, Korbo L, et al. Some new, simple and efficient stereological methods and their use in pathological research and diagnosis. *APMIS*. 1988;96(5):379-394.
